# Effective coverage of antenatal care and associated factors among pregnant women in Tanzania: a multilevel analysis

**DOI:** 10.3389/fgwh.2025.1477666

**Published:** 2025-08-15

**Authors:** Amanuel Yosef Gebrekidan, Beshada Zerfu Woldegeorgis, Gizachew Ambaw Kassie, Kirubel Eshetu Haile, Ashenafi Teklay Abrha, Angwach Abrham Asnake, Yordanos Sisay Asgedom

**Affiliations:** ^1^ School of Public Health, College of Health Sciences and Medicine, Wolaita Sodo University, Wolaita Sodo, Ethiopia; ^2^ Department of Internal Medicine, College of Health Sciences and Medicine, Wolaita Sodo University, Wolaita Sodo, Ethiopia; ^3^ School of Nursing, College of Health Sciences and Medicine, Wolaita Sodo University, Wolaita Sodo, Ethiopia; ^4^ Prevention Case Team, Subuha Seasie Woreda Health Office, Edagahamus, Tigray, Ethiopia

**Keywords:** antenatal care, effective coverage, multilevel analysis, quality, Tanzania

## Abstract

**Background:**

Antenatal care (ANC) is a crucial part of reproductive health care, providing opportunities for health promotion, screening, diagnosis, and illness prevention. However, evidence has shown that poor-quality ANC is prevalent. Therefore, this study aimed to investigate the effective coverage (quality-adjusted coverage) of ANC and its associated factors among pregnant women in Tanzania.

**Methods:**

This research was based on data from the 2022 Tanzania Demographic and Health Survey. The study utilized a weighted sample of 3,890 pregnant women. Given the influence of clustering and the binary nature of the outcome variable, we used a multilevel binary logistic regression model. Statistical significance was determined using the adjusted odds ratio (AOR) with a 95% confidence interval (CI), taking into account the model with the lowest deviation that best matched the data.

**Results:**

In this study, the effective coverage of ANC was 39.3% [95% confidence interval (CI): 37.8, 40.8]. After considering both individual- and community-level variables, women's age, educational status, husbands’/partners’ employment status, wealth index, number of ANC visits, administrative zones, and urban residence were all found to have statistically significant associations with effective ANC coverage among pregnant women in Tanzania.

**Conclusion:**

Only four out of ten pregnant women received effective ANC (quality-adjusted ANC), underscoring that crude coverage and access to healthcare do not ensure quality ANC. Emphasis should be placed on integrating component-based indicators. Furthermore, all attempts to provide all components should be considered during women's first visit, in addition to the need to increase the number of visits by pregnant women. Additionally, more attention should be paid to disadvantaged groups in terms of wealth and residence, and the fee exemption strategy should be supported by boosting the availability of healthcare supplies, particularly in remote areas.

## Introduction

In September 2015, the United Nations adopted the Sustainable Development Goals (SDGs), which reiterated the reduction of maternal and infant mortality as a global priority in the coming decades. Under SDG Goal 3, SDG Target 3.1 aims to reduce the global maternal mortality ratio (MMR) to less than 70 maternal deaths per 100,000 live births by 2030 (1). Between 1990 and 2015, the global maternal mortality ratio decreased by almost half. However, only nine countries with a maternal mortality ratio greater than 100 have met the Millennium Development Goals (MDG) 5 objective of a 75% reduction. Additionally, maternal mortality ratios rose in 12 nations, including the United States of America, while 26 countries did not make any progress (2).

Globally, progress in lowering maternal mortality during the MDG era slowed in the first five years of the SDG era (2016–2020). In 2020, the global MMR was estimated to be 223 deaths per 100,000 births. Over 287 000 women worldwide died from maternal causes, which equates to nearly 800 fatalities every day or one every two minutes (3). In 2020, Sub-Saharan Africa had the highest MMR, with a predicted 545 deaths per 100,000 live births (3). Tanzania is one of the countries in sub-Saharan Africa with the highest MMR, with a 2015/16 population-based survey revealing that the predicted average MMR in Tanzania was 556 per 100,000 live births. However, the MMR in the 2022 Tanzania Demographic and Health Survey and Malaria Indicator Survey (TDHS-MIS) was predicted to be 104 deaths per 100,000 live births, still far from the SDG target of 70 deaths per 100,000 live births by 2030 (4–6). To achieve this, one strategic outcome of the fifth Tanzanian Health Sector Strategic plan (2021–2026) is to reduce maternal and neonatal morbidity and mortality through the provision of equitable access to health and nutrition services. One stated focus of the strategic plan is to improve the quality of care in reproductive, maternal, newborn, child, and adolescent health to ensure maternal and child survival (7).

Antenatal care (ANC) is a crucial part of reproductive health care, providing opportunities for health promotion, screening, diagnosis, and illness prevention. It has been demonstrated that ANC can save lives by applying evidence-based practices in a timely manner and at the appropriate frequency (8). Infectious diseases in pregnancy, including syphilis, Human Immunodeficiency Virus (HIV), and malaria, as well as non-communicable illnesses like hypertension, diabetes, and pre-eclampsia, are all preventable risk factors for stillbirths, as well as complications for the birthing parent and the neonate that can be detected and managed by proper ANC (8).

Effective coverage (quality-adjusted coverage) of ANC can be understood as the proportion of pregnant women who had ANC visits and received the World Health Organization-recommended quality ANC, which includes providing caring and respectful evidence-based care, including examinations (e.g., history taking and physical assessments), diagnostic tests (e.g., blood sample test or urine sample test), preventive and curative treatments (e.g., tetanus vaccinations or iron supplementation), and counseling and education (e.g., on healthy eating or identifying potential complications) (8, 9). Three variables are usually used to assess the effective coverage (quality-adjusted coverage) of ANC: the number of contacts, timing of the first visit (before 12 weeks), and provision of all recommended services (8, 10).

Although maternal mortality and morbidity are high in Africa, findings from studies in Africa suggest that women of reproductive age have limited access to healthcare or face significant barriers to access. In sub-Saharan Africa, 61.5% of women experience barriers to healthcare access (11). Despite access to healthcare services being a problem in low- and middle-income countries (LMICs), challenges related to the quality of care also undermine efforts to expand access to critical services (12). Improving maternal health entails ensuring effective coverage and quality care for all women. Despite significant progress in extending access to health services in LMICs, the quality of care varies among nations and health conditions, hindering progress towards better health outcomes (13–15). Furthermore, better structural quality alone may not lead to improved service delivery or client satisfaction (16).

Different studies conducted in different countries have shown that poor-quality ANC is prevalent. Globally, 72·9% of women who received ANC underwent blood pressure monitoring, urine, and blood sample tests. This proportion varied from 6·3% in Burundi to 100% in Belarus (17). In low-income nations, 86·6% of women received ANC, whereas only 53·8% reported effective coverage of all three services (17). The process quality of ANC was 2.53%, 6.71%, 17.91%, 34.46%, and 48.07% in Uganda, Malawi, Rwanda, Kenya, and Namibia, respectively (18). While the process quality of ANC was 19.83% in Tanzania (18).

Different factors were found to be associated with the effective coverage of ANC. These factors included educational status, marital status, employment, wealth status, parity, number of ANC follow-ups, and type of health institution. Media exposure and residence were community-level variables associated with ANC quality (17, 19–21).

Most studies use crude coverage indicators, frequently used in maternal health studies, representing the tip of the iceberg, because they only consider contact between women and healthcare providers. They cannot show the level of adherence to care standards, the manner in which services are delivered, or the quality of care offered. In contrast, effective coverage combines the contact between service users and health services with the quality of care received, providing a proxy estimate of the potential desired healthcare outcomes through the use of services (22, 23). Although there are few studies on the quality of ANC in Tanzania, the majority of the evidence comes from previous demographic and health surveys or from studies conducted on a small scale that cannot represent the entire country, with an additional lack of identification of national-level determinant variables. Moreover, the few previous studies that assessed effective ANC coverage used data from the previous TDHS 2015/16 survey (9, 17), which may not reflect the current trend in the provision of effective ANC in Tanzania.

Therefore, this study aimed to use the most recent 2022 Tanzania Demographic and Health Survey and Malaria Indicator Survey (2022 TDHS-MIS) data to investigate the effective coverage of ANC and the factors associated with it among pregnant women in Tanzania, with the goal of providing baseline information on the national-level effective coverage (quality-adjusted coverage) of ANC and its determinants, thereby facilitating evidence-based decision-making and supporting the monitoring and evaluation of the Health Sector Strategic Plan V implementation.

## Methods and materials

### Study design and study area

We analyzed cross-sectional data from the 2022 Tanzania Demographic and Health Survey and Malaria Indicator Survey (2022 TDHS-MIS). According to Tanzania's 2022 population and housing census, the country has a total population of 61.7 million, with 59,851,347 living on the mainland and 1,889,773 in Zanzibar. Rural and urban areas account for 40.2 million and 21.5 million of the total population, respectively (24). Of the total population, 31.7 million were women and the remaining 30 million were men (24). The 2022 TDHS-MIS is the seventh DHS survey in Tanzania, conducted by the Health Surveys Program. Demographic and Health Surveys were conducted in 1991–1992, 1996, 1999, 2004–2005, 2010, and 2015–2016. The 2022 TDHS-MIS aims to provide current and credible data on population and health issues (4). The survey included a nationally representative sample of 16,354 households (4). Tanzania was divided into nine administrative zones to assess geographical disparities in population indicators. The Reproductive and Child Health Section of the Ministry of Health uses this classification scheme, despite the fact that these zones are not formally administrative areas. Grouping regions into zones leads to larger denominators and lower sampling errors for zonal indicators (4).

### Data source, population and sampling procedure

This analysis was based on the most recent 2022 TDHS-MIS, which was used to update health- and healthcare-related indicators for the country. The 2022 TDHS-MIS sample design was divided into two stages, with estimates for the total country, urban and rural areas in Tanzania Mainland, and Zanzibar (4). The first stage entailed selecting sampling points (clusters) from enumeration areas (EAs) chosen for the 2012 Tanzania Population and Housing Census (PHC). EAs were selected at a rate proportional to their size within each stratum. A total of 629 clusters were identified in this study. Of the 629 EAs, 211 were from urban areas and 418 from rural areas (4). The second stage consisted of choosing 26 households from each cluster, yielding a total sample size of 16,354 households for the 2022 TDHS-MIS (4).

This study utilized data from children under five years of age from the interviewed women's dataset (TZKR data). The investigation comprised 3,890 weighted samples of women who gave birth and had at least one ANC visit (live birth or stillbirth) within the two years prior to the survey.

### Study variables and measurement

The outcome variable of this study was the effective coverage (quality-adjusted coverage) of antenatal care (ANC). The outcome variable was binary, coded as 1 for receiving all seven necessary ANC components and 0 otherwise. The outcome variable was created according to WHO ANC guidelines. The WHO ANC guidelines suggest 49 recommendations grouped into five interventions: nutritional interventions, maternal and fetal assessment, preventive measures, interventions for common physiological symptoms, and health system interventions to improve the utilization and quality of ANC (8). However, owing to the vast nature of the recommended interventions and the lack of data on most of the recommended interventions, we opted to include seven common interventions based on data availability and previously published studies. These indicators only show the process dimension of quality. During each ANC visit, pregnant mothers should have their blood pressure measured, blood and urine samples taken, their baby's heartbeat checked, receive counseling about maternal diet, receive counseling about breast feeding, and be asked about vaginal bleeding. Each component provides a binary response (1 = yes, 0 = no) (8, 17, 20).

Given the hierarchical nature of the DHS data, we considered individual and community-level factors as independent variables. Individual-level variables included women's age, marital status, women's educational level, husband's/partner's educational level, women's occupation, wealth index, and distance to health facility. Household wealth indices are scored based on the number and type of consumer goods owned, such as televisions, bicycles, or vehicles, as well as living conditions, such as drinking water sources, bathroom facilities, and flooring materials (4). These scores were generated using principal component analysis. The national wealth quintiles were determined by assigning a household score to each household member, ranking each person based on their score, and dividing the distribution into five equal categories, each representing 20% of the population. We classified wealth status as poorest, poorer, middle, richer, and richest (4). Respondents were questioned subjectively about the distance to a health facility, and the response options were either a big problem or not a big problem (25). Maternal obstetric characteristics: Parity was classified as low if the mother had given birth once, multiparous if the mother had two to four births, and grand multiparous if the mother had five or more births (26). The number of ANC visits was classified as less than four visits and four visits and above. The timing of the first ANC was classified as less than 4 months, 4–6 months, and ≥7 months (25). The birth order of the last child was classified as first, second to fourth, and fifth or more (20). Community-level variables included residence classified as urban or rural, administrative zones, and community-level media exposure. Administrative zones: Tanzania was divided into nine zones to assess geographical disparities in population indicators. The Ministry of Health's Reproductive and Child Health Section employs this classification scheme, despite the fact that these zones are not formal administrative areas (4). When regions are divided into zones, the denominators become larger and the sample errors for zonal indicators decrease. Mainland Tanzania was divided into eight administrative zones: Western zone (Tabora and Kigoma), Northern zone (Kilimanjaro, Tanga, and Arusha), and Central zone (Dodoma, Singida, and Manyara). Southern Highlands zone: Iringa, Njombe, Ruvuma; Southern zone: Lindi, Mtwara; Southwest Highlands zone: Mbeya, Rukwa, Katavi, Songwe; Lake zone: Kagera, Mwanza, Geita, Mara, Simiyu, Shinyanga; Eastern zone: Dar es Salaam, Pwani, Morogoro. Zanzibar was grouped as a single zone (1 zone). Zanzibar Zone: Kaskazini Unguja, Kusini Unguja, Mjini Magharibi, Kaskazini Pemba, and Kusini Pemba (4). Community media exposure was categorized as “yes” if a household had access to one or more of the three media (read a newsletter, listen to the radio, and watch television) at least once a week, and “no” if a household had less than once a week or no access to the three media (reading a newsletter, listening to the radio, and watching television) (27).

### Data processing and analysis

Owing to the hierarchical nature of the data (individuals were nested within communities), and the ICC was 31%, a two-level mixed-effects logistic regression model was used to estimate both individual and community-level variables (fixed and random effects) on the effective coverage of ANC, and the log of the probability of the effective coverage of ANC was calculated. Weighted by sampling weight, primary sampling unit, and strata to account for sampling design and restore survey representativeness, we utilized a multilevel binary logistic regression model to evaluate the factors associated with the effective coverage of ANC, since the outcome variable was binary. Because DHS data are hierarchical, women are nested inside clusters; therefore, subjects within the same cluster may have comparable features to those in another cluster, thereby failing to meet the independence and equality of variance criteria. Consequently, an advanced multilevel cumulative logit model was used to account for the heterogeneity of the clusters. Four multilevel binary logistic regression models were created to assess the extent of the cluster variation in the quality of ANC. These models contained a null model that included no explanatory variables. The second model was adjusted for individual-level characteristics, and the third was adjusted for community-level characteristics of the respondents. The fourth model was fitted with variables at both the individual and community levels. To choose the best-fitted model for the data, model fitness was evaluated using deviance [−2 Log-Likelihood Ratio (LLR)], with the model with the lowest deviation being the most suitable. In the bivariable multilevel binary logistic regression model, variables with a *p*-value ≤ 0.2 were included in the multivariable analysis. Multivariate analysis was used to calculate the adjusted odds ratio (AOR) with a 95% confidence interval. Variables with a *p*-value < 0.05 were considered significantly associated with the effective ANC coverage.

To compare the differences between clusters, measures of variation (random effects) were reported using intra-class correlation coefficients (ICC), median odds ratios (MOR), and proportionate changes in variance (PCV). The ICC quantifies the similarity of observations within a cluster, whereas the MOR evaluates unexplained cluster heterogeneity (28). MOR is defined as the median odds ratio between the highest- and lowest-risk locations when two areas are randomly selected (28). In this study, the MOR indicates the extent to which a residential cluster affects the likelihood of effective ANC coverage. PCV assesses the overall variation induced by both individual- and area-level components in multilevel models (28). Multicollinearity was assessed using the variance inflation factor (VIF), and a cutoff point of greater than 5 was used to detect multicollinearity (29).

### Ethical consideration

As our study was a secondary analysis of publicly available survey data from the MEASURE DHS program, no ethical approval or participant agreement was required for this study. Following consent to use, the dataset was obtained from the Demographic and Health Survey Program website (https://dhsprogram.com). Furthermore, the dataset contained no personally identifiable information such as names or household numbers.

## Results

### Socio-demographic characteristics of respondents

This study used a weighted sample of 3,890 women who had been pregnant and had given birth within the previous two years (Table 1). The mean age of the women was 28.17 (SD ± 6.9) years, with over half (56.94%) married and 54.61% having a primary education. More than one-third (34.43%) of mothers were not working at the time of data collection, and 41.36% of households fell into the poorer and poorest wealth categories (Table 1).

**Table 1:** Sociodemographic and economic characteristics of study participants for effective coverage of antenatal care and associated factors among pregnant women in Tanzania.

Variable	Category	Weighted frequency	Percent
Women's age (in years)	15–19	395	10.15
	20–24	1,061	27.27
	25–29	975	25.06
	30–34	691	17.77
	35–39	511	13.14
	40–44	212	5.44
	45–49	45	1.17
Marital status	Never in a union	349	8.98
	Married	2,215	56.94
	Living with partner	993	25.53
	Widowed	30	0.76
	Divorced	179	4.6
	Separated/no longer living together	124	3.19
Women's education level	No education	771	19.82
	Primary	2,125	54.61
	Secondary	943	24.25
	Higher	51	1.31
Husband's/partner's education level (*n* = 3,208)	No education	447	13.93
	Primary	1,906	59.41
	Secondary	731	22.78
	Higher	124	3.87
Women's occupation	Not employed	1,339	34.43
	Employed	2,551	65.57
Husband's/partner's occupation (*n* = 3,208)	Employed	253	7.9
	Not employed	2,955	92.1
Wealth index	Poorest	842	21.65
	Poorer	767	19.71
	Middle	755	19.4
	Richer	777	19.99
	Richest	749	19.25
Distance to a health facility	Big problem	1,302	33.48
	Not a big problem	2,588	66.52

### Women's obstetrics-related characteristics

Of the 3,890 previously pregnant women, 45.15% had low parity. More than one-fourth of the mothers (27.48%) had less than four ANC visits, with 38.28 of all the respondents having their first ANC visit in less than four months of their pregnancy (Table 2).

**Table 2:** Pregnant women's obstetrics-related characteristics for effective coverage of antenatal care and associated factors among pregnant women in Tanzania.

Variable	Category	Weighted frequency	Percent
Parity	Low parity	1,756	45.15
	Multiparous	1,195	30.71
	Grand multiparous	939	24.14
Number of ANC visits	Less than four visits	1,069	27.48
	Four visits and above	2,821	72.52
Timing of first ANC in months	Less than 4 months	1,489	38.28
	4–6 months	2,138	54.97
	7 months and above	263	6.75
Birth order	1	906	23.3
	2–4	2,045	52.56
	5 and above	939	24.14
Number of under 5 children	0–1	1,556	40
	2–3	2,049	52.67
	Four and above	285	7.33

### Community-level characteristics

Regarding community-level characteristics, the vast majority of the study participants (71.83%) lived in urban areas, with over one-third (34.48%) living in the Lake Administrative Zone. More than half of the households (57.65%) lacked media exposure (Table 3).

**Table 3:** Community-level variables for effective coverage of antenatal care and associated factors among pregnant women in Tanzania.

Variable	Category	Weighted frequency	Percent
Residence	Urban	1,096	28.17
	Rural	2,794	71.83
Administrative zones	Western	371	9.54
	Northern	366	9.42
	Central	416	10.7
	Southern highlands	208	5.35
	Southern	172	4.42
	South west highlands	350	8.98
	Lake	1,341	34.48
	Eastern	541	13.9
	Zanzibar	125	3.21
Community level media exposure	Yes	1,647	42.35
	No	2,243	57.65

### Effective coverage of ANC among pregnant women in Tanzania

**Figure 1:**
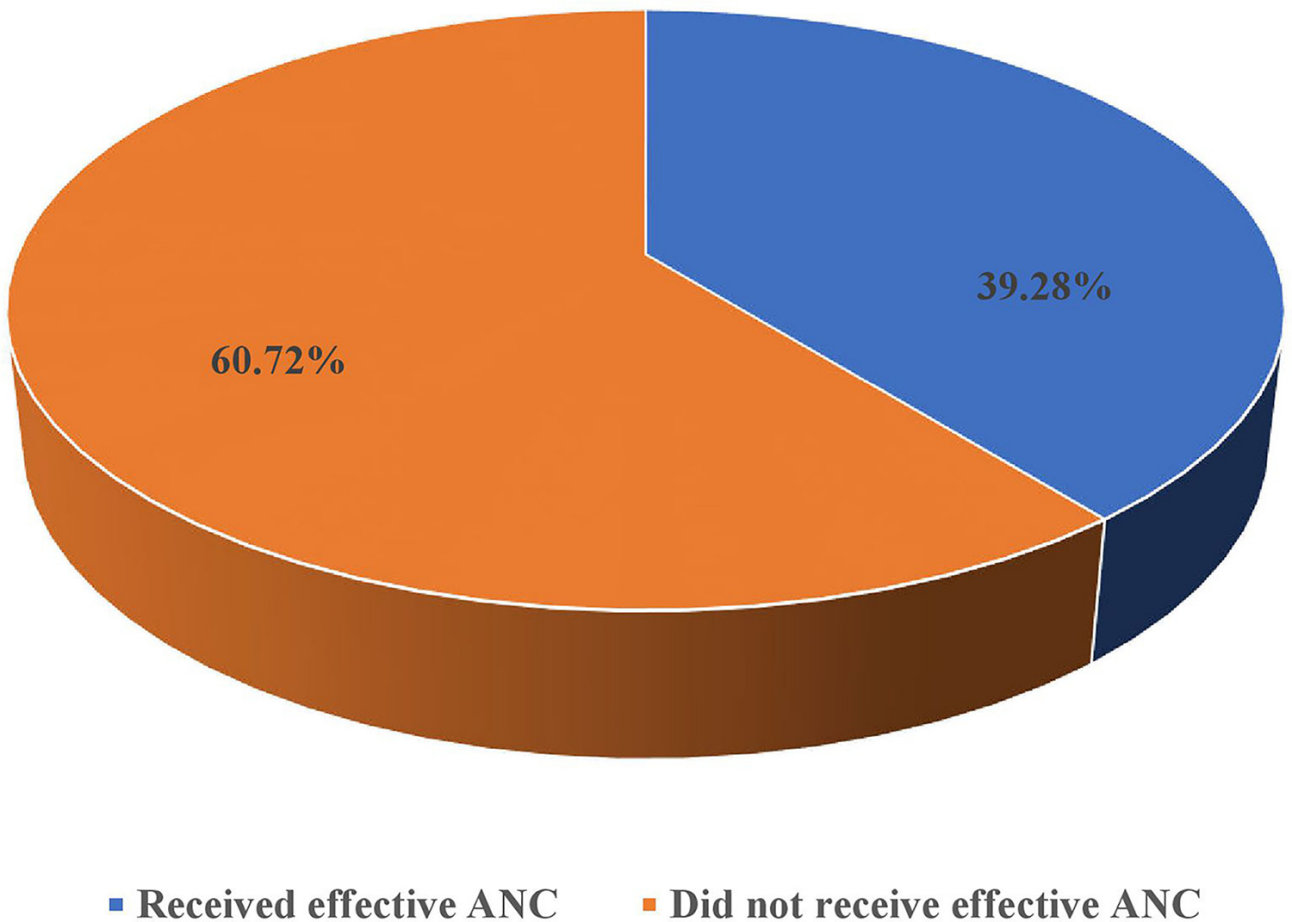
The proportion of effective coverage of ANC among pregnant womenwith ANC visits in Tanzania.

Of the 3,890 pregnant women with at least one ANC visit, only 1,528 (39.28%) received effective ANC coverage in Tanzania. The proportion of effective coverage was 39.28% [95% CI: 37.76, 40.83] (Figure 1). The most common service provided was listening to a baby's heartbeat, with 95.81% of pregnant women having their babies heartbeats listened to, while only 66.58% were asked about vaginal bleeding (Table 4). A total of 25 (0.64%) pregnant women did not receive any of the seven ANC components during pregnancy.

**Table 4:** Provision of ANC components among pregnant women in Tanzania.

Component of ANC	Category	Frequency	Percentage
Blood pressure taken	Yes	2,966	76.23
	No	924	23.77
Urine sample taken	Yes	2,682	68.94
	No	1,208	31.06
Blood sample taken	Yes	3,557	91.42
	No	333	8.58
Listen to baby's heartbeat	Yes	3,727	95.81
	No	163	4.19
Nutritional counseling	Yes	2,688	69.11
	No	1,202	30.89
Breast feeding counseling	Yes	2,616	67.24
	No	1,274	32.76
Asked about vaginal bleeding	Yes	2,590	66.58
	No	1,300	33.42

### Random effect analysis (measures of variation)

#### Null model (model 1)

This model had no predictors. We evaluated the likelihood ratio to determine whether the multilevel binary logistic regression model was more significant than the single-level binary logistic regression model. The LR test results were significant (*p* < 0.01), indicating that the multilevel binary logistic regression model best matched the results of the single-level binary logistic regression analysis. As a result, the LR test proposed using a multilevel binary logistic regression model. Four random-effects models were fitted, and the final model was selected based on the lowest deviation.

The ICC of the null model was 31%, indicating that the cluster variation explained 31% of the overall variability in the quality of ANC. Furthermore, the median odds ratio (MOR) for effective ANC coverage was 2.38, indicating variation between clusters. If we chose pregnant women at random from two distinct clusters, those in the cluster with higher effective coverage of ANC had a 2.38 times higher likelihood of receiving effective ANC than those in the cluster with lower effective coverage. The best-fit model was selected based on the lowest deviation value (3507.56) (Table 5).

**Table 5:** Multilevel analysis of factors associated with the effective coverage of antenatal care among pregnant women in Tanzania.

Variables	Received Quality ANC care		COR (95% CI)	Model 1 AOR (95% CI) (With level one variables)	Model 2 AOR (95% CI) (With level two variables)	Model 3 AOR (95% CI) (With level one and two variables)
	Yes *n* (%)	No *n* (%)				
Individual level variables						
Women's age (in years)						
15–19	135 (34.15)	260 (65.85)	1	1		1
20–24	350 (32.96)	711 (67.04)	0.89 (0.66, 1.21)	0.89 (0.61, 1.29)		0.86 (0.6, 1.25)
25–29	450 (46.2)	525 (53.8)	1.57 (1.16, 2.13)	1.57 (1.05, 2.35)		1.46 (0.97, 2.18)
30–34	262 (37.95)	429 (62.05)	1.15 (0.84, 1.58)	1.4 (0.88, 2.21)		1.25 (0.79, 1.99)
35–39	227 (44.42)	284 (55.59)	1.54 (1.1, 2.16)	2.13 (1.29, 3.54)		1.77 (1.07, 2.95)*
40–44	94 (44.42)	118 (55.58)	1.63 (1.07, 2.47)	2.66 (1.48, 4.77)		2.14 (1.19, 3.85)*
45–49	10 (21.78)	35 (78.22)	0.56 (0.24, 1.33)	0.97 (0.34, 2.75)		0.77 (0.27, 2.18)
Women's educational status						
No education	186 (24.17)	585 (75.83)	1	1		1
Primary education	786 (37)	1,339 (63)	1.46 (1.16, 1.83)	1.31 (1.01, 1.71)		1.25 (0.96, 1.63)
Secondary education	513 (54.39)	430 (45.61)	2.5 (1.92, 3.28)	1.65 (1.18, 2.32)		1.46 (1.03, 2.06)*
Higher education	42 (83.26)	9 (16.74)	15.24 (6.42, 36.18)	5.57 (2.05, 15.12)		4.72 (1.74, 12.8)**
Husbands’/partners’ educational level (*n* = 3,208)						
No education	118 (26.28)	329 (73.72)	1	1		1
Primary education	677 (35.54)	1,229 (64.46)	1.07 (0.8, 1.43)	0.88 (0.65, 1.19)		0.86 (0.64, 1.17)
Secondary education	386 (52.75)	345 (47.25)	2.01 (1.44, 2.81)	1.13 (0.78,1.64)		1.12 (0.77, 1.62)
Higher education	77 (61.9)	47 (38.1)	3.09 (1.83, 5.25)	0.85 (0.47, 1.55)		0.97 (0.53, 1.76)
Husbands’/partners’ occupation (*n* = 7,799)						
Not employed	68 (26.88)	185 (73.12)	1	1		1
Employed	1,189 (40.25)	1,766 (59.75)	1.92 (1.35, 2.75)	1.71 (1.18, 2.46)		1.59 (1.1, 2.3)*
Wealth index						
Poorest	211 (25.05)	631 (74.95)	1	1		1
Poorer	208 (27.09)	559 (72.91)	1.09 (0.83, 1.42)	1.03 (0.76, 1.39)		1.01 (0.74, 1.36)
Middle	254 (33.63)	501 (66.37)	1.42 (1.08, 1.86)	0.99 (0.72, 1.36)		0.83 (0.6, 1.16)
Richer	372 (47.81)	406 (52.19)	2.68 (2.02, 3.56)	1.63 (1.16, 2.29)		1.2 (0.83, 1.75)
Richest	484 (64.61)	265 (35.39)	5.07 (3.74, 6.88)	2.76 (1.87, 4.08)		1.72 (1.09, 2.71)*
Distance to a health facility						
Big problem	373 (28.66)	929 (71.34)	1	1		1
Not a big problem	1,155 (44.63)	1,433 (55.37)	1.73 (1.42, 2.1)	1.29 (1.03, 1.6)		1.23 (0.76, 1.65)
Parity						
Low parity	730 (41.55)	1,026 (58.45)	1.14 (0.93, 1.41)	1.29 (0.88, 1.89)		1.12 (0.76, 1.65)
Multiparous	505 (42.24)	690 (57.76)	1.28 (1.03, 1.6)	1.32 (0.98, 1.76)		1.19 (0.89, 1.6)
Grand multiparous	294 (31.27)	645 (68.73)	1	1		1
Number of ANC visits						
Less than four visits	274 (25.62)	795 (74.38)	1	1		1
Four visits and above	1,254 (44.46)	1,567 (55.54)	1.97 (1.63, 2.39)	1.6 (1.25, 2.05)		1.58 (1.23, 2.03)**
Timing of first ANC in months						
Less than 4 months	694 (46.61)	795 (53.39)	1	1		1
4- 6 months	775 (36.23)	1,364 (63.77)	0.75 (0.63, 0.89)	1.01 (0.83, 1.23)		1.01 (0.82, 1.23)
7 months and above	59 (22.61)	203 (77.39)	0.44 (0.3, 0.63)	0.78 (0.49, 1.25)		0.8 (0.5, 1.28)
Number of under five children						
0–1	727 (46.74)	829 (53.26)	1.64 (1.15, 2.35)	1.38 (0.91, 2.09)		1.09 (0.71, 1.66)
2–3	720 (35.12)	1,329 (64.88)	1.12 (0.79, 1.58)	0.96 (0.64, 1.44)		0.82 (0.54, 1.23)
Four and above	81 (28.45)	204 (71.57)	1	1		1
Community level variables						
Administrative zones						
Western	64 (17.24)	307 (82.76)	1		1	1
Northern	180 (49.24)	186 (50.76)	5.6 (3.09, 10.16)		4.95 (2.87, 8.53)	4.3 (2.38, 7.77)**
Central	148 (35.49)	269 (64.51)	2.61 (1.44, 4.73)		2.74 (1.59, 4.71)	2.5 (1.39, 4.49)**
Southern highlands	145 (69.63)	63 (30.37)	14.58 (7.59, 27.99)		12.05 (6.57, 22.09)	11.7 (5.94, 23.1)**
Southern	87 (50.73)	85 (49.27)	6.42 (3.23, 12.76)		6.54 (3.48, 12.03)	5.14 (2.52, 10.46)**
South West highlands	121 (34.57)	229 (65.43)	2.43 (1.35, 4.4)		2.14 (1.24, 3.69)	2.17 (1.2, 3.92)*
Lake	428 (31.89)	914 (68.11)	1.93 (0.1.14, 3.26)		1.73 (1.07, 2.8)	1.74 (1.03, 2.91)*
Eastern	281 (51.88)	260 (48.12)	6.49 (3.68, 11.47)		3.59 (2.11, 6.11)	3.29 (1.84, 5.88)**
Zanzibar	75 (59.88)	50 (40.12)	8.59 (4.42, 16.7)		7.1 (3.81, 13.26)	4.41 (2.22, 8.75)**
Community level media exposure						
Yes	815 (49.5)	832 (50.5)	1.69 (1.42, 2.01)		1.47 (1.23, 1.74)	1.1 (0.89, 1.36)
No	713 (31.78)	1,530 (68.22)	1		1	1
Residence						
Urban	630 (57.5)	466 (42.5)	3.75 (2.85, 4.94)		3.08 (2.36, 4.02)	1.93 (1.37, 2.73)**
Rural	898 (32.13)	1,896 (67.87)	1		1	1
Measures of variation						
	Null model		Model 1		Model 2	Model 3
Variance	1.48		1.04		0.83	0.82
ICC	0.31		0.24		0.20	0.19
AIC	4873.46		3660.6		4482.98	3581.57
BIC	4885.97		3824.58		4558.06	3806.27
PCV	Ref.		29.73%		43.92%	44.59%
MOR 2.38 [95% CI: 2.06, 2.69]						
Model fitness (Goodness of fit)						
LLR	−2434.73		−1803.3		−2229.49	−1753.78
Deviance (− 2LLR)	4869.46		3606.6		4458.98	3507.56
LR-test	368.58		217.58		261.9	175.33

AIC, Akaike information criteria; AOR, adjusted odds ratio; BIC, Bayesian information criteria; ICC, intra-class correlation coefficient; LLR, log-likelihood ratio; LR, likelihood ratio; MOR, median odds ratio; PCV, proportionate change in variance.

* *P*-value <0.05.

** *P*-value <0.01.

#### Factors associated with effective coverage of ANC among pregnant mothers in Tanzania

Bivariate analysis was applied to identify candidate variables for the multilevel multivariable mixed-effects binary logistic regression. Women's age, women's educational status, husbands'/partners' educational status, husbands'/partners' occupation, wealth index, distance to a health facility, parity, number of ANC visits, timing of the first ANC, number of children under 5 years, administrative zones, community-level media exposure, and residence were all associated with the effective coverage of ANC (*p* value < 0.2). Birth order was excluded from the analysis due to multicollinearity. Women's age, women's educational status, husbands'/partners' occupation, wealth index, number of ANC visits, administrative zones, and residence were statistically significant factors associated with the effective coverage of ANC among pregnant women in Tanzania (*p*-value < 0.05).

Pregnant women aged 30–35 and 40–45 were 1.77 times [AOR = 1.77, 95% CI: 1.07, 2.95] and 2.14 times [AOR = 2.14, 95% CI: 1.19, 3.85] more likely to receive effective ANC than those aged 15–19 years, respectively. The odds of receiving effective ANC among pregnant women with secondary and higher education were 1.46 times [AOR = 1.46, 95% CI: 1.03, 2.06] and 4.72 times [AOR = 4.72, 95% CI: 1.74, 12.8] higher than those among pregnant women with no education. Pregnant women with an employed husband/partner had 1.59 times higher odds [AOR = 1.59, 95% CI: 1.1, 2.3] of receiving effective ANC than those with an unemployed husband/partner. Considering the wealth index, pregnant women from the richest wealth quantile were 1.72 times [AOR = 1.72, 95% CI: 1.09, 2.71] more likely to receive effective ANC than those in the poorest quantile. Furthermore, pregnant women with four or more ANC visits were 1.58 times [AOR = 1.58, 95% CI: 1.23, 2.03] more likely to receive effective ANC than those with less than four ANC visits. With respect to administrative zones, the odds of receiving effective ANC among pregnant women residing in the Northern, Central, Southern highlands, Southern, Southwest highlands, Lake, Eastern, and Zanzibar administrative zones were 4.3 times [AOR = 4.3, 95% CI: 2.38, 7.77], 2.5 times [AOR = 2.5, 95% CI: 1.39, 4.49], 11.7 times [AOR = 11.7, 95% CI: 5.94, 23.1], 5.14 times [AOR = 5.14, 95% CI: 2.52, 10.46], 2.17 times [AOR = 2.17, 95% CI: 1.2, 3.92], 1.74 times [AOR = 1.74, 95% CI: 1.03, 2.91], 3.29 times [AOR = 3.29, 95% CI: 1.84, 5.88], and 4.41 times [AOR = 4.41, 95% CI: 2.22, 8.75] higher, respectively, compared to pregnant women residing in the Western administrative zone. Moreover, the odds of effective ANC among pregnant women living in urban areas were 1.93 times [AOR = 1.93, 95% CI: 1.37, 2.73] higher than those among pregnant women living in rural areas (Table 5).

## Discussion

This study used the most recent 2022 Tanzania Demographic and Health Survey and Malaria Indicator Survey (2022 TDHS-MIS) data to investigate the status and factors associated with the effectiveness of ANC among pregnant women in Tanzania. The study found that the effective coverage of ANC among pregnant women who had ANC visits in Tanzania was only 39.28% [95% CI: 37.76, 40.83].The most common service provided was listening to a baby's heartbeat, with 95.81% of pregnant women having their babies heartbeat listened to. This was followed by blood sample collection (91.42%), blood pressure measurement (76.23%), nutritional counseling (69.11%), urine sample collection (68.94%), breast feeding counseling (67.24%), and being asked about vaginal bleeding (66.58%). A total of 25 (0.64%) pregnant women did not receive any of the seven ANC components during pregnancy. After considering both individual and community-level variables, women's age, educational status, husbands'/partners' occupation, wealth index, number of ANC visits, administrative zones, and residence were all found to have statistically significant associations with effective coverage of ANC among pregnant women in Tanzania.

In this study, only 39.3% [95% CI: 37.8, 40.8] of pregnant women in Tanzania received effective ANC; this finding is lower than a multilevel analysis conducted in Ghana, which reported an effective ANC coverage of 61% (30), and a study that included 91 national household surveys, which reported an effective ANC coverage of 53.8% in low-income countries and 72.9% overall (17). One possible explanation for this is the inclusion of different parameters to operationalize ANC quality. This study used seven ANC components, while the study that assessed 91 national household surveys used only three components (blood pressure monitoring and blood and urine testing) to operationalize ANC quality (17). As the number of components necessary to operationalize the effective coverage of ANC increases, the status of effective coverage of ANC is expected to decline. Furthermore, the inclusion of middle-income nations, which account for 61 of the 91 included countries, will increase the proportion of effective ANC coverage, as there is a clear relationship between gross domestic product (GDP) per capita and effective coverage of ANC, which may explain the effective coverage of ANC gap between Ghana and Tanzania, as Ghana's GDP per capita is nearly double that of Tanzania (17, 31).

In contrast, the finding of this study is higher than those of studies conducted in nine East African countries (9), East Africa (32), Central and West Africa (33), and Ethiopia (20), with reported proportions of effective ANC coverage of 21%, 11.16%, 29.35%, and 22.48%, respectively. This could possibly be explained by the survey years included, in which the listed studies were conducted from 2008 to 2018, while the current study used the most recent 2022 TDHS-MIS data and advancements, and the more focus given to maternal and child health in recent years with the increasing economic growth observed in Tanzania might affect the discrepancies observed. Moreover, this discrepancy could be attributed to the inclusion of multiple nations with significant differences in prior studies. This could be due to unequal access to ANC services (20, 34). Additionally, the current study included women with a live or stillbirth in the two years preceding the survey, while the other studies included women who gave birth within the five years preceding the survey. This may have caused recall bias, reducing the proportion of effective ANC coverage.

This study also identified significant individual and community-level variables associated with effective ANC coverage. Women's age was associated with effective ANC coverage. Pregnant women aged 30–35 and 40–45 were 1.77 and 2.14 times more likely to receive effective ANC than those aged 15–19 years. This finding is in line with studies conducted in East Africa, Nigeria, and Ghana that found that as age increased, the odds of receiving quality ANC also increased (32, 35, 36). Older mothers may be more aware of the value of using ANC services. Additionally, older mothers may have received additional health education and counseling during previous pregnancies, increasing their understanding of the benefits of ANC (32). This might indicate that parity and the number of children might predict the possibility of having effective ANC. However, our study found no association between parity or the number of children and effective ANC. Further research is necessary to understand the justification for older age and effective ANC.

The odds of receiving effective ANC among pregnant women with secondary and higher education were 1.46 times higher than those among pregnant women with no education. This finding is also reported in studies conducted in Tanzania (37), Ethiopia (20), East Africa (32), Ghana (36), three African countries (38), and low- and middle-income countries (39). One possible explanation for the observed discrepancy is that educated women are more aware of the procedures to expect during a prenatal visit and are therefore more inclined to request them. Poor education, in particular, may cause a social distance between the patient and the healthcare provider, making effective communication difficult. Furthermore, women with low levels of education may be treated differently in health facilities, contributing to inequalities in education. Women with less education may face discrimination from service providers who do not provide thorough information about pregnancy care or perform all necessary tests (38).

Pregnant women with employed husbands/partners had 1.59 times higher odds of receiving effective ANC than those with unemployed husbands/partners. This finding is similar to a study conducted in Nepal, where a husband's occupation was associated with four or more ANC follow-ups (40). This might be due to the increased probability of frequent follow-ups and the probability of accessing those services at least once during follow-ups.

Regarding the wealth index, pregnant women from the richest wealth quantile were 1.72 times more likely to receive effective ANC than those in the poorest quantile. The association between wealth status and the quality of ANC provided has also been reported in studies conducted in Ethiopia (20), Nine East African countries (9), East Africa (32), Nigeria (35), West and Central Africa (33), Nepal (40), and low- and middle-income countries (17, 39). This could be the result of access to maternal and reproductive health care being influenced by economic circumstances, which in turn can be associated with media exposure and travel time to the institution, all of which impact the quality of ANC (41, 42). Rich women may have greater access to ANC information through the mass media. Traveling to distant health facilities indirectly increases the cost of ANC, making it more affordable for pregnant women from wealthy homes compared to their peers (43). Despite Tanzania's fee exemption policy, which allows mothers to receive maternal health services for free, implementing the user fee exemption has been difficult. The primary hurdles to accessing maternal health services were increased costs and health commodity shortages. Furthermore, both pregnant women and health care professionals indicated that a lack of health commodities and insufficient service providers resulted in extended wait times and hampered access to health commodities (44). The evidence suggests that poor individuals face not only limited coverage but also low-quality services.

Furthermore, pregnant women with four or more ANC visits were 1.58 times more likely to receive effective ANC than those with less than four ANC visits. This finding is consistent with those reported from Rwanda (45), Ethiopia (20), and Nigeria (35). This might be due to the fact that women who have four or more ANC visits have more contact with healthcare providers, which increases their chances of receiving comprehensive health education and all ANC components. Furthermore, four or more ANC visits can be considered indicative of a woman's awareness of and devotion to her well-being during pregnancy (45, 46). This underscores the importance of considering a pregnant woman's first ANC visit as a last resort, as well as the need to provide all ANC quality components at the first contact.

Moreover, the odds of receiving effective ANC among pregnant women residing in the Northern, Central, Southern highlands, Southern, Southwest highlands, Lake, Eastern, and Zanzibar administrative zones were higher than those among pregnant women residing in the Western administrative zone. This could possibly be explained by the wealth and socioeconomic differences in the administrative zones as well as the GDP per capita difference among the administrative zones, where the two regions of Tabora and Kigoma, which are ranked in the lowest five regions by GDP per capita, are included under the Western administrative zone compared to other administrative zones (47). Despite the fact that quality ANC services in Tanzania remain poor, more focus should be paid to the Western administrative zone's regions.

Additionally, the odds of receiving effective ANC were 1.93 times higher among pregnant women in urban areas than pregnant women living in rural areas. This is also supported by studies conducted in Ethiopia (32, 48), Nigeria (35), West Africa (33), and Nepal (40). Rural areas may have less developed health infrastructure and fewer skilled healthcare staff, which could explain this finding (35). This could also be related to the fact that rural areas have much less or possibly no transportation infrastructure, making access to health care difficult, further limiting the number of ANC visits, and reducing the probability of receiving all ANC components (40, 49).

### Limitations

Despite the fact that this study used a weighted pooled nationally representative TDHS survey in Tanzania with a sample size large enough to detect the true effect of the independent variables, as well as multilevel binary logistic regression analysis to achieve credible estimates and standard errors, the use of a cross-sectional study design cannot establish a causal relationship between the effective coverage of ANC and independent variables. In addition, owing to the nature of the data, this study did not assess the structural and outcome components of quality. Furthermore, because it relies on respondents' answers, the study may have been influenced by recall bias.

## Conclusion

In Tanzania, effective coverage (quality-adjusted coverage) of ANC only covers four out of ten pregnant women, demonstrating that crude coverage and access to health care do not ensure quality ANC. Women's age, educational status, husbands'/partners' occupation, wealth index, number of ANC visits, administrative zones, and residence were all found to be statistically significant predictors of effective ANC coverage. The findings highlight the significant gap between service contacts and effective service provision, which must be bridged to improve maternal and newborn health and reduce morbidity and mortality from maternal and neonatal health-related conditions, as stated in Tanzania's Health Sector Strategic Plan V. The findings support Tanzania's Health Sector Strategic Plan V, which aims to improve the quality of maternal and newborn healthcare to ensure maternal and child survival. As a result, rather than focusing solely on analyzing ANC crude coverage and utilization, an emphasis on effective coverage of ANC is necessary. Because quality improvement is a long-term effort, the Ministry of Health should allocate resources to both short-term gains and long-term reforms. Regional/district health management teams should focus on short-term wins, including increasing the frequency of ANC visits to increase the likelihood of providing recommended services to pregnant women, integrating component-based indicators, and raising awareness among ANC service providers to consider that every pregnant woman's ANC visit can be her last. The Ministry of Health and regional health management teams should also work on long-term reforms and prioritize strengthening the fee exemption strategy, which should be supported by investing in the expansion and improvement of healthcare infrastructure, such as counseling rooms and examination equipment, particularly in remote areas and where the economically disadvantaged reside. Furthermore, emphasis should be placed on implementing a robust monitoring and evaluation system to ensure that quality standards are met.

## Data Availability

Publicly available datasets were analyzed in this study. This data can be found here: https://dhsprogram.com.

## References

[1] Program UND. Sustainable Development Goals in Action Geneva. (2015). Available online at: https://www.undp.org/geneva/sustainable-development-goals/good-health (Accessed March 17, 2024).

[2] World Health Organization. Trends in Maternal Mortality: 1990–2015: Estimates from WHO, UNICEF, UNFPA, World Bank Group and the United Nations Population Division. Geneva: World Health Organization (2015).

[3] WHO U, UNFPA, World Bank Group and UNDESA/Population Division. Trends in Maternal Mortality 2000 to 2020: Estimates by WHO, UNICEF, UNFPA, World Bank Group and UNDESA/Population Division. Geneva: WHO (2023).

[4] Ministry of Health (MoH) [Tanzania Mainland] MoHMZ, National Bureau of, Statistics (NBS) OotCGSO, and ICF. Tanzania Demographic and Health Survey and Malaria Indicator Survey 2022 Final Report. Dodoma, Tanzania, and Rockville, Maryland, USA: MoH, NBS, OCGS, and ICF (2022).

[5] Ministry of Health CD, Gender, Elderly and Children (MoHCDGEC) [Tanzania, Mainland] MoHMZ, National Bureau of Statistics (NBS), Office of the Chief, Government Statistician (OCGS) aI. Tanzania Demographic and Health Survey and Malaria Indicator Survey (TDHS-MIS) 2015–16. Dar es Salaam, Tanzania, and Rockville, Maryland, USA: MoHCDGEC, MoH, NBS, OCGS, and ICF (2016).

[6] Network HN. Maternal Mortality Tanzania 2000-2020. (2023). Available online at: https://www.healthynewbornnetwork.org/resource/maternal-mortality-tanzania-2000-2020/ (Accessed March 19, 2024).

[7] Ministry of Health CD, Gender, Elderly and Children. Health Sector Strategic Plan July 2021—June 2026. (2021).

[8] Organization WH. WHO recommendations on antenatal care for a positive pregnancy experience. (2016). 28079998

[9] Bobo FT Asante A Woldie M Hayen A. Poor coverage and quality for poor women: inequalities in quality antenatal care in nine East African countries. Health Policy Plan. (2021) 36(5):662–72. 10.1093/heapol/czaa192 33822943

[10] Heredia-Pi I Servan-Mori E Darney BG Reyes-Morales H Lozano R. Measuring the adequacy of antenatal health care: a national cross-sectional study in Mexico. Bull W H O. (2016) 94(6):452. 10.2471/BLT.15.168302 27274597 PMC4890208

[11] Seidu AA. Mixed effects analysis of factors associated with barriers to accessing healthcare among women in sub-Saharan Africa: insights from demographic and health surveys. PLoS One. (2020) 15(11):e0241409. 10.1371/journal.pone.0241409 33166320 PMC7652334

[12] Das J. The quality of medical care in low-income countries: from providers to markets. PLoS Med. (2011) 8(4):e1000432. 10.1371/journal.pmed.1000432 21532744 PMC3075231

[13] Victora CG Requejo JH Barros AJ Berman P Bhutta Z Boerma T Countdown to 2015: a decade of tracking progress for maternal, newborn, and child survival. Lancet. (2016) 387(10032):2049–59. 10.1016/S0140-6736(15)00519-X 26477328 PMC7613171

[14] Kruk ME Gage AD Arsenault C Jordan K Leslie HH Roder-DeWan S High-quality health systems in the sustainable development goals era: time for a revolution. Lancet Glob Health. (2018) 6(11):e1196–e252. 10.1016/S2214-109X(18)30386-3 30196093 PMC7734391

[15] Koblinsky M Moyer CA Calvert C Campbell J Campbell OM Feigl AB Quality maternity care for every woman, everywhere: a call to action. Lancet. (2016) 388(10057):2307–20. 10.1016/S0140-6736(16)31333-2 27642018

[16] Do M Wang W Hembling J Ametepi P. Quality of antenatal care and client satisfaction in Kenya and Namibia. Int J Qual Health Care. (2017) 29(2):183–93. 10.1093/intqhc/mzx001 28453821

[17] Arsenault C Jordan K Lee D Dinsa G Manzi F Marchant T Equity in antenatal care quality: an analysis of 91 national household surveys. Lancet Glob Health. (2018) 6(11):e1186–e95. 10.1016/S2214-109X(18)30389-9 30322649 PMC6187112

[18] Owili PO Muga MA Mendez BR Chen B. Quality of care in six sub-Saharan Africa countries: a provider-based study on adherence to WHO’s antenatal care guideline. Int J Qual Health Care. (2019) 31(1):43–8. 10.1093/intqhc/mzy105 30428045

[19] Afulani PA Buback L Essandoh F Kinyua J Kirumbi L Cohen CR. Quality of antenatal care and associated factors in a rural county in Kenya: an assessment of service provision and experience dimensions. BMC Health Serv Res. (2019) 19:1–16. 10.1186/s12913-019-4476-4 31590662 PMC6781384

[20] Negash WD Fetene SM Shewarega ES Fentie EA Asmamaw DB Teklu RE Multilevel analysis of quality of antenatal care and associated factors among pregnant women in Ethiopia: a community based cross-sectional study. BMJ Open. (2022) 12(7):e063426. 10.1136/bmjopen-2022-063426 35902185 PMC9341179

[21] Young MR Morof D Lathrop E Haddad L Blanton C Maro G Beyond adequate: factors associated with quality of antenatal care in western Tanzania. Int J Gynaecol Obstet. (2020) 151(3):431–7. 10.1002/ijgo.13349 32799345 PMC8009327

[22] Ng M Fullman N Dieleman JL Flaxman AD Murray CJ Lim SS. Effective coverage: a metric for monitoring universal health coverage. PLoS Med. (2014) 11(9):e1001730. 10.1371/journal.pmed.1001730 25243780 PMC4171091

[23] Gebremedhin AF Dawson A Hayen A. Effective coverage of newborn postnatal care in Ethiopia: measuring inequality and spatial distribution of quality-adjusted coverage. PLoS One. (2023) 18(10):e0293520. 10.1371/journal.pone.0293520 37883459 PMC10602323

[24] The United Republic of Tanzania (URT) MoFaP, Tanzania National Bureau of Statistics and President’s Office—Finance and Planning, Office of the Chief Government Statistician, Zanzibar. The 2022 Population and Housing Census: Administrative Units Population Distribution Report. Tanzania. (2022).

[25] Croft TN Allen CK Zachary BW Guide to DHS Statistics. Rockville, Maryland, USA: ICF (2023).

[26] Amare T Sime T Legese GL Alemu MB. A multilevel analysis of factors associated with vitamin A supplementation among children aged 6–35 months in Ethiopia. Front Public Health. (2023) 11:1052016. 10.3389/fpubh.2023.1052016 36908452 PMC9995845

[27] Gebremedhin T Aschalew AY Tsehay CT Dellie E Atnafu A. Micronutrient intake status and associated factors among children aged 6–23 months in the emerging regions of Ethiopia: a multilevel analysis of the 2016 Ethiopia demographic and health survey. PLoS One. (2021) 16(10):e0258954. 10.1371/journal.pone.0258954 34679088 PMC8535338

[28] Hox J Moerbeek M Van de Schoot R. Multilevel Analysis: Techniques and Applications. New York, NY: Routledge (2017).

[29] Donath C Gräßel E Baier D Pfeiffer C Bleich S Hillemacher T. Predictors of binge drinking in adolescents: ultimate and distal factors-a representative study. BMC Public Health. (2012) 12:1–15. 10.1186/1471-2458-12-263 22469235 PMC3378431

[30] Afulani PA. Determinants of stillbirths in Ghana: does quality of antenatal care matter? BMC Pregnancy Childbirth. (2016) 16(1):132. 10.1186/s12884-016-0925-9 27255155 PMC4891927

[31] Bank TW. GDP per capita. (2022).

[32] Raru TB Mamo Ayana G Bahiru N Deressa A Alemu A Birhanu A Quality of antenatal care and associated factors among pregnant women in east Africa using demographic and health surveys: a multilevel analysis. Women’s Health. (2022) 18:17455065221076731. 10.1177/17455065221076731 PMC8819820 35114855

[33] Olorunsaiye CZ Brunner Huber L Laditka S Kulkarni SJ Boyd S. Individual and community socioeconomic factors related to the quality of antenatal care: a multilevel analysis of West and Central Africa. Women Health. (2021) 61(1):15–26. 10.1080/03630242.2020.1847748 33256565

[34] Tessema ZT Minyihun A. Utilization and determinants of antenatal care visits in East African countries: a multicountry analysis of demographic and health surveys. Adv Public Health. (2021) 2021. 10.1155/2021/6623009

[35] Fagbamigbe AF Idemudia ES. Assessment of quality of antenatal care services in Nigeria: evidence from a population-based survey. Reprod Health. (2015) 12(1):88. 10.1186/s12978-015-0081-0 26382228 PMC4574449

[36] Atinga RA Baku AA. Determinants of antenatal care quality in Ghana. Int J Soc Econ. (2013) 40(10):852–65. 10.1108/IJSE-2011-0075

[37] Rwabilimbo AG Ahmed KY Page A Ogbo FA. Trends and factors associated with the utilisation of antenatal care services during the millennium development goals era in Tanzania. Trop Med Health. (2020) 48(1):38. 10.1186/s41182-020-00226-7 32518496 PMC7268642

[38] Babalola S. Women’s education level, antenatal visits and the quality of skilled antenatal care: a study of three African countries. J Health Care Poor Underserved. (2014) 25(1):161–79. 10.1353/hpu.2014.0049 24509018

[39] Amo-Adjei J Aduo-Adjei K Opoku-Nyamah C Izugbara C. Analysis of socioeconomic differences in the quality of antenatal services in low and middle-income countries (LMICs). PLoS One. (2018) 13(2):e0192513. 10.1371/journal.pone.0192513 29474362 PMC5825027

[40] Joshi C Torvaldsen S Hodgson R Hayen A. Factors associated with the use and quality of antenatal care in Nepal: a population-based study using the demographic and health survey data. BMC Pregnancy Childbirth. (2014) 14(1):94. 10.1186/1471-2393-14-94 24589139 PMC3943993

[41] Sanogo N Yaya S. Wealth status, health insurance, and maternal health care utilization in Africa: evidence from Gabon. BioMed Res Int. (2020) 2020:4036830. 10.1155/2020/4036830 32461984 PMC7212326

[42] Ahmed S Creanga AA Gillespie DG Tsui AO. Economic status, education and empowerment: implications for maternal health service utilization in developing countries. PLoS One. (2010) 5(6):e11190. 10.1371/journal.pone.0011190 20585646 PMC2890410

[43] Rahman M Haque SE Mostofa MG Tarivonda L Shuaib M. Wealth inequality and utilization of reproductive health services in the Republic of Vanuatu: insights from the multiple indicator cluster survey, 2007. Int J Equity Health. (2011) 10:1–10. 10.1186/1475-9276-10-58 22132828 PMC3286423

[44] Mollel D Kagashe GA Asingizwe D Banzimana S Maru SM Niragire F. Barriers to access of maternal health commodities among pregnant women in public health facilities in ubungo municipal council, Tanzania. J Pharm Policy Pract. (2024) 17(1):2300457. 10.1080/20523211.2023.2300457 38234995 PMC10793628

[45] Sserwanja Q Nuwabaine L Gatasi G Wandabwa JN Musaba MW. Factors associated with utilization of quality antenatal care: a secondary data analysis of Rwandan demographic health survey 2020. BMC Health Serv Res. (2022) 22(1):812. 10.1186/s12913-022-08169-x 35733151 PMC9217119

[46] Sserwanja Q Nabbuye R Kawuki J. Dimensions of women empowerment on access to antenatal care in Uganda: a further analysis of the Uganda demographic health survey 2016. Int J Health Plann Manage. (2022) 37(3):1736–53. 10.1002/hpm.3439 35178763

[47] Statista. Gross Domestic Product per capita at current prices in Tanzania in 2020, by region 2020. Available online at: https://www.statista.com/statistics/1149410/gdp-per-capita-at-current-prices-in-tanzania-by-region/ (Accessed April 12, 2024).

[48] Shiferaw K Mengistie B Gobena T Dheresa M Seme A. Extent of received antenatal care components in Ethiopia: a community-based panel study. Int J Women’s Health. (2021) 13:803–13. 10.2147/IJWH.S327750 34526826 PMC8435480

[49] Teijlingen E Simkhada P Stephens J Simkhada B Rogers S Sharma S. Making the best use of all resources: developing a health promotion intervention in rural Nepal. Health Renaissance. (2012) 10(3):229–35. 10.3126/hren.v10i3.7141

